# Tetratricopeptide repeat domain 9A is an interacting protein for tropomyosin Tm5NM-1

**DOI:** 10.1186/1471-2407-8-231

**Published:** 2008-08-12

**Authors:** Shenglan Cao, Gay Hui Ho, Valerie CL Lin

**Affiliations:** 1 School of Biological Sciences, Nanyang Technological University, Singapore; 2 Department of Surgical Oncology, National Cancer Center, Singapore

## Abstract

**Background:**

Tetratricopeptide repeat domain 9A (TTC9A) protein is a recently identified protein which contains three tetratricopeptide repeats (TPRs) on its C-terminus. In our previous studies, we have shown that TTC9A was a hormonally-regulated gene in breast cancer cells. In this study, we found that TTC9A was over-expressed in breast cancer tissues compared with the adjacent controls (P < 0.00001), suggesting it might be involved in the breast cancer development process. The aim of the current study was to further elucidate the function of TTC9A.

**Methods:**

Breast samples from 25 patients including the malignant breast tissues and the adjacent normal tissues were processed for Southern blot analysis. Yeast-two-hybrid assay, GST pull-down assay and co-immunoprecipitation were used to identify and verify the interaction between TTC9A and other proteins.

**Results:**

Tropomyosin Tm5NM-1 was identified as one of the TTC9A partner proteins. The interaction between TTC9A and Tm5NM-1 was further confirmed by GST pull-down assay and co-immunoprecipitation in mammalian cells. TTC9A domains required for the interaction were also characterized in this study. The results suggested that the first TPR domain and the linker fragment between the first two TPR domains of TTC9A were important for the interaction with Tm5NM-1 and the second and the third TPR might play an inhibitory role.

**Conclusion:**

Since the primary function of tropomyosin is to stabilize actin filament, its interaction with TTC9A may play a role in cell shape and motility. In our previous results, we have found that progesterone-induced TTC9A expression was associated with increased cell motility and cell spreading. We speculate that TTC9A acts as a chaperone protein to facilitate the function of tropomyosins in stabilizing microfilament and it may play a role in cancer cell invasion and metastasis.

## Background

Human tetratricopeptide repeat domain 9 (TTC9) was first reported as a hypothetical protein KIAA0227 by Nagase et al, based on the sequence analysis of a cDNA clone isolated from a brain cDNA library [1]. It was later identified as a steroid hormone-regulated gene in various breast cancer cells [2]. It seems that there is a family of TTC9 protein. The MGC program at the National Institutes also identified cDNA sequences named as TTC9B and TTC9C, which share 46% and 35% homology with TTC9A in amino acids sequence, respectively [3]. However, TTC9B and TTC9C have not been identified at the protein level. Nonetheless, to keep up with the information in the NCBI database, TTC9 is now referred to as TTC9A in this article.

TTC9 family belongs to a large family of tetratricopeptide repeat (TPR)- containing proteins. The TPR domain is a 34 amino acids (aa) consensus motif that is found in tandem repeats of varying number in different proteins [4-6]. Circular dichroism (CD) studies indicate that TPR motifs are approximately 50% α-helical structures with little or no β-sheet formation [7]. Crystallographic structure analysis of TPR-containing proteins revealed that TPR motif generally forms an antiparallel α-helical hairpin [8,9]. Clustering of these hairpins in tandem generates a domain with a grooved surface and dimension that can conveniently grasp another polypeptide. Generally, by generating a flexible, mutable domain that can facilitate specific protein-protein interactions, the TPR motif presents an elegant evolutionary solution contributing to the fundamental biological importance of coordinating interactions among gene products [6]. The functions of TPR containing protein include cell cycle control [10], transcription and splicing events [11], protein transport especially protein import [12], regulatory phosphate turnover [13], and protein folding [14]. TTC9A contains three TPR domains at its carboxyl-terminus, at amino acid positions 57–90, 128–161 and 164–197.

In previous studies, we have identified the open reading frame of TTC9 gene and confirmed the protein size of TTC9A to be 222 aa. Using mouse polyclonal antibody generated against TTC9A protein, TTC9A was shown to be ubiquitously expressed in human tissues. In breast cancer cells, TTC9A was predominantly concentrated to the endoplasmic reticulum and was regulated by a number of factors, such as growth factors, serum factors and steroid hormones [2]. Although existing results suggest that TTC9A could be an important protein ubiquitously expressed in all cell and tissue types, the exact role of TTC9A remains unclear.

In this study, we found that TTC9A mRNA was significantly over-expressed in breast cancer tissues compared with the adjacent normal breast tissues, which suggested TTC9A could be an important gene involved in hormone signaling and breast cancer development. By yeast-two-hybrid assay, we identified one of TTC9A interacting proteins, TM5/TM30nm, which is also referred to as Tm5NM-1 and is a non-muscle tropomyosin encoded by γ-tropomyosin gene. The tropomyosins are a group of actin-binding proteins found in skeletal muscle, smooth muscle and non-muscle tissues. They are either hetero- or homo-dimeric proteins with a rod-shaped, α-helical coiled-coil structure. Usually the dimers form a head-to-tail polymer running along the major groove in the actin filament [15,16]. Mammalian and avian tropomyosins are encoded by four genes, i.e. α, β, γ, δ [17]. Historically, the tropomyosin proteins have been divided into two classes, high-molecular-weight (HMW) and low-molecular- weight (LMW), which are ~284 aa and 247 aa in length, respectively. This size difference is generated by the use of alternative promoters [17,18], alternative splicing of mRNA [19], and different 3' UTR processing [20]. These mechanisms give rise to over 40 tropomyosin isoforms.

Tropomyosins are believed to be involved in the stabilization of actin filaments [21]. Actin filaments which lack tropomyosins tend to be rapidly undergoing assembly and disassembly process, such as those associated with neuronal growth cone filopodia and the leading edge of mammary adenocarcinoma cells [19,22-24]. In skeletal muscle, tropomyosins serve to mediate the effect of Ca^2+ ^on the actin-myosin interaction [25,26]. Instead of binding Ca^2+ ^directly, they perform this function by acting as bifunctional molecules, binding to actin on one hand, and providing specific sites for the binding of the troponin complex of regulatory proteins on the other hand [27-30]. Though the precise function of non-muscle tropomyosin is less understood, some in vitro studies have shown that non-muscle tropomyosins are able to differentially protect actin from the severing action of gelsolin [31] and can regulate the Mg-ATPase activity of myosins to varying degrees [32]. Furthermore, it seems that non-muscle tropomyosins play an important role in tumor development. For example, down-regulation of tropomyosin 2 was essential in ras-mediated malignant transformation of fibroblasts [33,34]. Tropomyosin 3 isoform 2 (also termed as TM5/TM30nm or Tm5NM-1) was expressed at a higher level in highly metastatic B16 mouse cell line than in mouse cell line exhibiting a lower metastasis rate. Similar result was also obtained in rat cells [35,36].

This study also identified specific domains in TTC9A that is crucial in the interaction with Tm5NM-1. The significance of the interaction between TTC9A and Tm5NM-1 is yet to be elucidated. On the other hand, our previous studies have shown that in breast cancer cells, the up-regulation of TTC9A expression by progesterone was associated with increased cell focal adhesion and motility. All these findings suggest a possible role of TTC9A in cell-matrix adhesion and in tropomyosin-mediated stabilization of actin microfilament.

## Methods

### Cell line and reagents

COS-7 cells were obtained from Dr. Koh Cheng Gee, School of Biological Sciences, Nanyang Technological University, Singapore.

COS-7 cells were routinely maintained in phenol red- and high D-glucose- containing Dulbecco's Modified Eagle Medium (DMEM) supplemented with 7.5% fetal calf serum (FCS), 2 mM glutamine and 40 mg/L gentamicin.

All cell culture reagents were bought from Invitrogen (Carlsbad, California). Fetal calf sera were from Hyclone (Logan, UT) or PromoCell GmbH (Heidelberg, Germany). All cell culture plastic wares were purchased from Falcon (Becton Dickinson, San Jose, CA), NUNC (Nalge Nunc International, Rochester, NY), or Corning (Corning, NY).

### TTC9A expression in breast cancer tissues

Human tissue samples were obtained from the Tissue Repository at the National Cancer Centre (NCC), Singapore. Tissue samples were harvested at the time of mastectomy or breast conserving surgery with prior signed informed consent from the patients. Matched pairs of malignant tissue and the adjacent normal breast tissue were harvested and confirmed histologically by a pathologist and were snap frozen in liquid nitrogen. The cases utilized in this study were collected between January 2002 and December 2003. Clinicopathological data such as tumor size, nuclear grade and hormone receptor status were obtained from a prospective database. This study was approved by the ethics committee at NCC.

25 matched pairs of breast tissues were mashed using a mortar and pestle. Total RNA was extracted using TRIzol reagent (Life Technologies Inc.) according to the manufacturer's instructions. 5 μg of total RNA from each sample was reverse transcribed using Superscript II reverse transcriptase (Invitrogen). 1 μl cDNA produced from each RT reaction was amplified by PCR. The primers used here were 5'-CACAT GTCTATAACGATTTCC-3' (forward) and 5'-TGCAGGAAACAGGGG ACTCTC-3' (reverse). 10 μl PCR products corresponding to individual breast tissue sample were separated on 1% agarose gel and transferred to nylon Hybond-N membrane (Amersham Biosciences). ^32^P-labeled TTC9A were generated by random priming reaction (Amersham Biosciences) using the same PCR product of TTC9A. The band intensities were analyzed using Bio-Rad Molecular Image Analyzer. As internal controls, 36B4 and GAPDH genes were also included for normalization. The primers used to amplify 36B4 gene were 5'-GATTGGCTACCCAACTGTTGCA-3' (forward) and 5'-CAGGGGCAG CAGCCACAAAGGC-3' (reverse). The primers for GAPDH were 5'-TGCACCACCA ACTGCTTAG-3' (forward) and 5'-GAGGCAGGGATGATG TTC-3' (reverse).

### Yeast-two-hybrid assay screen

Yeast-two-hybrid assay screen was carried out using MATCHMAKER LexA Two-Hybrid System from Clontech Laboratories Inc (Mountain View, CA) according to the manufacturer's instructions. Yeast *Saccharomyces cerevisiae *MATα strain EGY48 was transformed with lacZ reporter gene, bait plasmid containing TTC9A coding sequence (TTC9A-pLexA) and cDNA libraries of human breast cancer cell line MCF-7 (OriGene Technologies, Inc., Rockville, MD) using the lithium acetate method [37]. Transformed EGY48 was plated onto SD/Gal/Raf/-His/-Trp/-Ura/-Leu+X-Gal plates. An interaction was considered positive when two reporter genes, LEU2 and *lac*Z, were activated. The interactions were further verified by co-immunoprecipitation or GST pull-down assay.

### Transfection of COS-7 cells

COS-7 cells were seeded the day before transfection. The confluence of cells at the time of transfection was 40%–50%. Transfection was carried out with FuGENE 6 Transfection Reagent (Roche Diagnostics, Basel, Switzerland) according to the manufacturer's instructions. Cells were harvested at 48 hours post-transfection. The expression of protein was examined by Western blotting.

### Cell lysates preparation and protein concentration quantification

Cells were lysed on ice with cold lysis buffer containing 100 mM NaF, 50 mM HEPES (pH7.5), 150 mM NaCl, 1% Triton X-100, 1 mM PMSF and the cocktail of proteinase inhibitors (5 μg/ml pepstatin A, 5 μg/ml leupeptin, 2 μg/ml aprotinin and 1 mM Na3VO4). The cell debris was discarded by centrifuging at 14,000 rpm for 12 min at 4°C. The supernatants were immediately frozen down in liquid nitrogen and were stored at -80°C for future use. Protein concentrations were determined by BCA protein assay kit (Pierce, Rockford, IL).

### Purification of GST-TTC9A

The 669 bp TTC9A coding sequence was cloned into pGEX-5X-3 (Amersham Biosciences) for the expression of GST-TTC9A protein. GST or GST-TTC9A protein was purified with Glutathione Sepharose 4B (Amersham Biosciences) according to the manufacturer's instructions. Eluted protein was pooled and dialyzed in PBS for future experiment.

### GST pull-down assay

COS-7 cells were transfected with Tm5NM-1-(His)_6 _expression vector or control vector (pcDNA3.1/myc-His(-) B) respectively and total cell lysates were collected at 48 h post-transfection. 60 μg GST-TTC9A protein was immobilized onto 12 μl Glutathione Sepharose 4B gel (Amersham Biosciences) by gentle rotation at 4°C for 2 h. 300 μg total cell lysates collected were then added and the total reaction volume was brought up to 1 ml by PBS. The reactions were incubated overnight at 4°C with gentle rotation. Nonspecific binding proteins were removed by washing in cold washing buffer containing 100 mM NaF, 50 mM HEPES (pH 7.5) and 150 mM NaCl for four times followed by one more wash in PBS. Proteins bound to the beads were eluted with 2 × SDS-PAGE sample buffer and were separated on an SDS-PAGE gel. Tm5NM-1-(His)_6 _protein was detected using anti-His antibody (Amersham Biosciences). GST protein expressed by empty pGEX-5X-3 vector was included as a negative control.

### Co-immunoprecipitation with anti-flag M2 Affinity Gel

Anti-flag M2 Affinity Gel was bought from Sigma-Aldrich (St. Louis, MO). Co-immunoprecipitation was carried out according to the manufacturer's instructions. Briefly, COS-7 cells were transfected with flag-TTC9A or flag-TTC9A fragments and Tm5NM-1-(His)_6 _expression vectors using FuGENE 6 Transfection Reagent. The amount of Tm5NM-1-(His)_6 _vector was two times more than those of flag-TTC9A or flag-TTC9A fragments vectors. 400 μg of total protein lysates collected were mixed with 15 μl anti-flag M2 Affinity Gel and the total reaction volume was brought up to 1 ml by lysis buffer. After incubating with the cell lysates overnight at 4°C with gentle rotation, the affinity gel was washed three times with 0.5 ml TBS. Proteins bound were eluted with 2 × SDS-PAGE sample buffer and were loaded onto an SDS-PAGE gel. Tm5NM-1-(His)_6 _protein was detected using anti-His antibody (Amersham Biosciences). COS-7 cells transfected with empty pXL-flag vector and Tm5NM-1-(His)_6 _expression vector were included as a negative control.

### Co-immunoprecipitation of endogenous Tm5NM-1 and TTC9A-flag

COS-7 cells were transfected with TTC9A-flag expression vector using FuGENE 6 Transfection Reagent according to the manufacturer's instructions. Cell lysates were collected at 48 h post-transfection. 500 μg of total protein lysates collected were mixed with 5 μl anti-Tm5NM-1 antibody (Chemicon International Inc., Temecula, CA) or goat pre-immune serum for 4 h at 4°C with gentle rotation. 40 μl protein A/G plus-agarose beads were then added and the mixture was incubated overnight with gentle rotation at 4°C. The agarose beads were washed four times with washing buffer containing 100 mM NaF, 50 mM HEPES (pH 7.5), 150 mM NaCl, 0.01% Triton X-100. Proteins bound were eluted with 2 × SDS-PAGE sample buffer and were loaded onto an SDS-PAGE gel. TTC9A-flag protein was detected using anti-flag antibody (Sigma-Aldrich).

### Statistical analysis

The experiment for TTC9A expression in human breast cancer tissues and adjacent normal tissues were analyzed by the Mann-Whitney nonparametric test using the SPSS program for Windows, version 11.5. Difference between the expression of TTC9A in normal and cancer tissues was considered as significant when the P value is less than 0.05.

## Results and Discussion

### TTC9A is over-expressed in breast cancer tissues compared with the adjacent normal tissues

**Figure 1:**
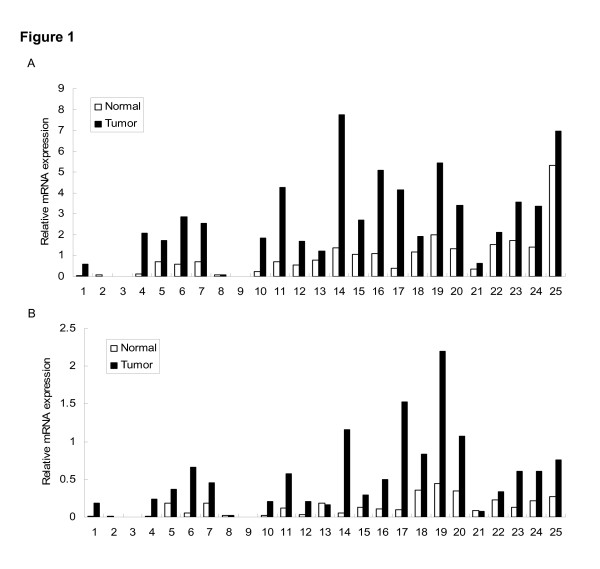
**The expression level of TTC9A mRNA was significantly higher in breast cancer tissues than that in the adjacent normal breast tissues.** Total RNA was extracted from human breast cancer tissues and the matched adjacent normal breast tissues. Equal amount of RNA from each sample was subjected to reverse transcription and cDNA produced was amplified by PCR using TTC9A, 36B4 or GAPDH primers. 10 μl PCR products were separated on an agarose gel and analyzed by Southern blotting. Band intensity was analyzed by Bio-Rad Molecular Image Analyzer. The figure shows the expression levels of TTC9A in 25 pairs of normal and tumor tissue samples after normalizing to those of 36B4 (A) or GAPDH (B). Each pair of bars represents samples from one patient. The primers used for TTC9A were 5'-CACATGTCTATAACGATTT CC-3' (forward) and 5'-TGCAGGAAACAGGGG ACTCTC-3' (reverse). The primers used to amplify 36B4 gene were 5'-GATTGGCTACCCAACTGTTGCA-3' (forward) and 5'-CAGGGGCAGCAGCCACAAAGGC-3' (reverse). The primers for GAPDH were 5'-TGCACCACCAACTGCTTAG-3' (forward) and 5'-GAGGCAGGGATGATG TTC-3' (reverse).

Previous results have shown that TTC9A is a hormonally regulated gene *in vitro *[2]. As breast cancer is well-known to be a hormone-dependent malignancy, it is noteworthy to know whether TTC9A is over-expressed in breast cancer tissues and if its expression is correlated with hormone receptor status. 25 matched pairs of human breast cancer tissue and the adjacent normal tissue were analyzed for TTC9A mRNA expression. The results presented in Fig. 1 were obtained by RT-PCR as illustrated in the "materials and methods" and the PCR products were quantitated by Southern blotting analysis. 36B4, which codes for human acidic ribosomal phosphoprotein P0, was used as a control for cDNA input (Fig. 1A). Since the expression of 36B4 varied among samples, GAPDH was also included as a normalization standard for cDNA input. It turned out that the expression levels of these housekeeping genes were always higher in tumor tissues compared with the corresponding normal tissues. This variation has been reported before. For example, GAPDH expression was 3.3-fold higher in seminoma compared with normal testis [38]. Similarly, GAPDH transcription was significantly greater in both colonic adenomas and cancers than in normal mucosa [39]. Nonetheless, the results revealed that the relative expression of TTC9A mRNA, when normalized to either 36B4 or GAPDH, was significantly higher in breast cancer tissue compared with its adjacent normal tissue (Fig. 1) (*P *< 0.00001). It is also notable that nearly all the tumor tissues expressed higher level of TTC9A mRNA compared with its adjacent controls. However, we found no correlation of TTC9A expression with other clinic pathological data such as tumor size, nuclear grade, axillary's lymph node status or hormone receptor expression.

### Yeast-two-hybrid assay identified Tm5NM-1 as one of TTC9A interacting proteins

The observation that TTC9A was over-expressed in breast cancer tissues suggests potential relevance of the protein in breast cancer biology. In an initial attempt to investigate the relationship between TTC9A function and cancer development, we searched for binding partners of the protein. To accomplish this, we performed a yeast-two-hybrid screening of a cDNA library from breast cancer cell line MCF-7, using full-length TTC9A as the bait. The coding region of the 222 aa TTC9A protein was cloned into the 'bait' vector, pLex A, which contains the DNA-binding domain. MCF-7 cDNA libraries were fused to the activation domain in the 'pray' vector p42AD. The bait vector and the cDNA libraries were transformed into yeast strain EGY48 together with a lacZ reporter gene. 30 positive clones, in which the coding regions of the library plasmids were in frame with the activation domain according to the sequencing results, were obtained after two rounds of specificity test. Among them, 5 clones contained genes coding for human tropomyosin Tm5NM-1, which is encoded by γ-tropomyosin gene of the tropomyosin family.

### TTC9A protein can interact with cellular expressed Tm5NM-1

**Figure 2:**
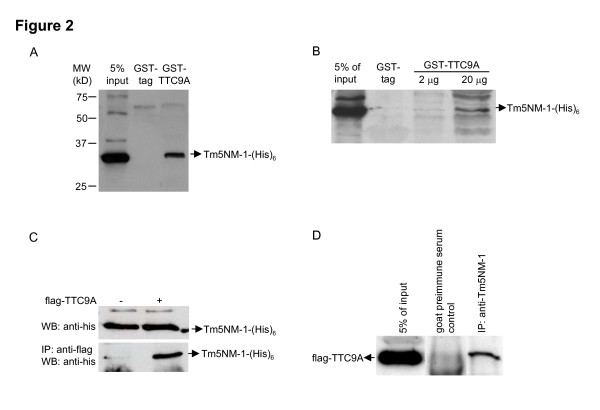
**TTC9A binds to Tm5NM-1.** (A) GST-TTC9A binds to Tm5NM-1-(His)_6_. COS-7 cells were transfected with Tm5NM-1-(His)_6 _expression vector or control vector and total cell lysates were collected at 48 h post-transfection. 60 μg GST-TTC9A protein was immobilized onto Glutathione Sepharose 4B gel (Amersham Biosciences) and 300 μg total cell lysates were used for Tm5NM-1-(His)_6 _pull-down. The proteins bound to the beads were eluted with 2 × SDS-PAGE sample buffer and were separated on an SDS-PAGE gel. Tm5NM-1-(His)_6 _was detected using anti-His antibody (Amersham Biosciences). GST protein expressed by empty pGEX-5X-3 vector was included as a negative control. 15 μg total cell lysates (5% of input) were loaded in the first lane to indicate the position of Tm5NM-1-(His)_6 _band. (B) Tm5NM-1-(His)_6 _pull-down by GST-TTC9A is concentration-dependent. GST-pull down assay was carried out with 2 μg or 20 μg GST-TTC9A as bait protein. The amount of Tm5NM-1-(His)_6 _pulled down was proportional to the amount of bait protein used. (C) TTC9A-flag interacted with Tm5NM-1-(His)_6_. Expression vectors for TTC9A-flag and Tm5NM-1-(His)_6 _were co-transfected into COS-7 cells. Co-immunoprecipitation was carried out with anti-flag agarose beads (Sigma-Aldrich) and Tm5NM-1 was detected by anti-His antibody (Amersham Biosciences). Upper panel: Tm5NM-1-(His)_6 _was expressed at similar level in control vector and TTC9A-flag transfected COS-7 cells; lower panel: Tm5NM-1-(His)_6 _was pulled down by TTC9A-flag. (D) TTC9A-flag interacted with endogenous Tm5NM-1-(His)_6_. Expression vector for TTC9A-flag was transfected into COS-7 cells. Co-immunoprecipitation was carried out with anti-Tm5NM-1/2 (Chemicon) antibody and TTC9A was detected by anti-flag antibody (Sigma-Aldrich). Co-immunoprecipitation with goat pre-immune serum was included as a negative control.

The binding of TTC9A to Tm5NM-1 was further examined by GST-pull down assay. In Fig. 2A, Tm5NM-1-(His)_6 _expression vector was transfected into COS-7 cells and GST-TTC9A was used as a "bait" to pull down the cellular expressed Tm5NM-1 protein. As shown in the figure, Tm5NM-1 was pulled down by GST-TTC9A, but not by the GST-tag, suggesting that GST-TTC9A did interact with cellular expressed Tm5NM-1. The specificity of the interaction was further confirmed by GST pull-down assay with different amount of bait protein. Fig. 2B showed that the protein amount of Tm5NM-1 pulled down by GST-TTC9A increased proportionally to the amount of bait protein used in the assay. However, bands other than the expected Tm5NM-1 were also observed in Fig. 2B and these non-specific bands were not observed in sample with GST protein. These unexpected bands could be due to non-specific pull-down or degraded GST-TTC9A.

### TTC9A interacts with Tm5NM-1 in mammalian cells

The interaction between Tm5NM-1 and TTC9A was further verified by co-immunoprecipitation in mammalian cells. As shown in Fig. 2C, cellular expressed TTC9A-flag pulled down prominent amount of Tm5NM-1-(His)_6 _protein in COS-7 cells. To further confirm the interaction, TTC9A-flag expression vector was transfected into COS-7 cells and the interaction between endogenous Tm5NM-1 and cellular expressed TTC9A was examined by co-immunoprecipitation with anti-Tm5NM-1 antibody. Fig. 2D revealed that cellular expressed flag-TTC9A could also bind to endogenous Tm5NM-1.

### Identification of the regions/domains in TTC9A which are important in the interaction with Tm5NM-1

**Figure 3:**
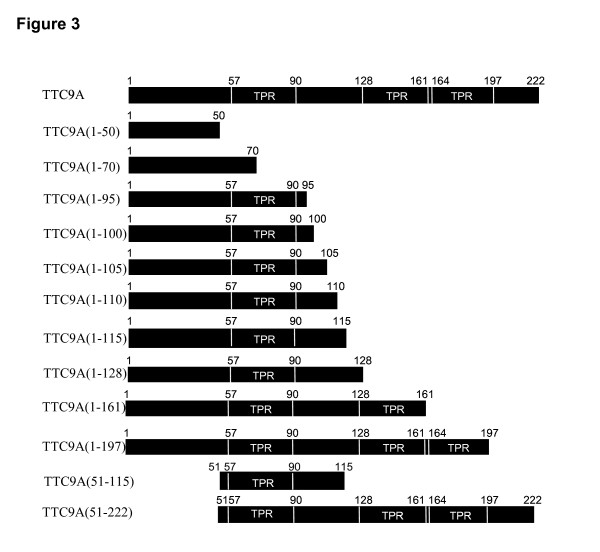
Truncation constructs of TTC9A protein and their relative binding to Tm5NM-1 based on co-immunoprecipitation experiment.

**Figure 4:**
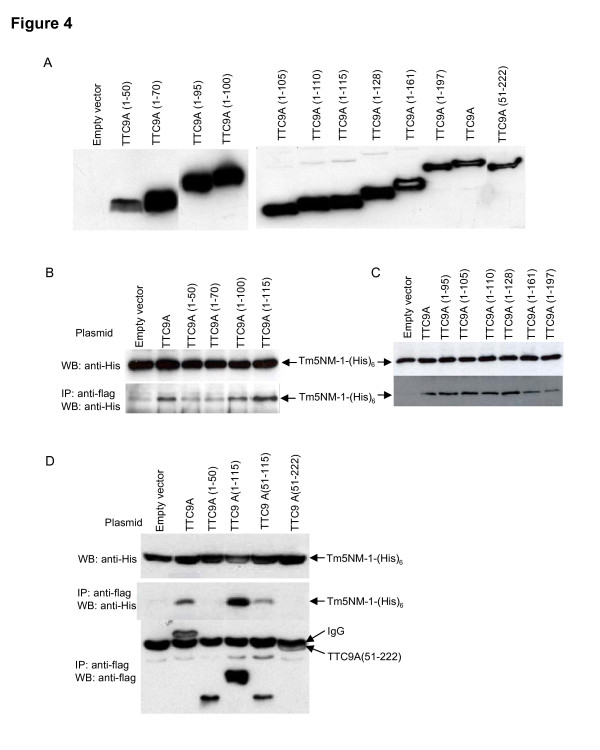
**Domains involved in TTC9A and Tm5NM-1 interaction.** (A) Western blot analysis of the expression TTC9A truncates in the cell lysates using anti-flag antibody from Sigma-Aldrich. All truncations expressed proteins of predicted sizes. The hollow streak in some of the bands indicates over-saturation of the signal. (B and C) Test of Tm5NM-1-(His)_6 _pull-down by different TTC9A truncations in pXL-Flag vectors. The TTC9 truncation constructs were transfected into COS-7 cells together with Tm5NM-1-(His)_6 _expression vector. The interaction between flag-TTC9A truncates and Tm5NM-1-(His)_6 _was analyzed by co-immunoprecipitation with anti-flag agarose beads (Sigma-Aldrich). The upper panels in B and C are Western blotting analysis of Tm5NM-1 expression in the cell lysates using anti-His antibody (Amersham Biosciences), and the lower panels are the Tm5NM-1-(His)_6 _co-immunoprecipitated with flag-TTC9A and the truncated proteins. (D) The upper panel is the analysis of Tm5NM-1 expression in the cell lysates; the middle panel is the co-immunoprecipitated Tm5NM-1 with flag-TTC9A and the truncated proteins; the lower panel represents the amount of TTC9A and its truncates pulled down by the anti-flag agarose beads (Sigma-Aldrich) in the co-immunoprecipitation assay.

To specify the domains that interact with Tm5NM-1, different truncations of TTC9A protein shown in Fig. 3 were constructed and tested in COS-7 cells. As is shown in Fig. 4A, all truncations were expressed at the expected protein sizes. It is to be noted that the whitish streak in the centre of the bands for TTC9A (1–161), TTC9A (1–197), full length TTC9A and TTC9A (51–222) were due to the over-saturation of the signal. This means that the amounts of protein expressed by these constructs were not necessarily less than those by other smaller truncations which showed broader bands. The reason may be that in 12% gel, the smaller proteins tend to be more diffuse resulting in a broader band, whereas higher molecular weight proteins tend to be more compact in migration.

Fig. 4B showed that TTC9A (1–50) and TTC9A (1–70) did not pull-down Tm5NM-1 visibly as compared with vector-transfected control, regardless of the very high expression level of TTC9A (1–70). TTC9A (1–100), which contains the first TPR domain, interacted with Tm5NM-1 to some extent but the interaction was weaker than TTC9A (1–115). Since the first TPR domain lies in residues 57–90, it is plausible that the first TPR domain is required for the interaction. To confirm this postulation, more truncations of TTC9A protein were tested. Fig. 4C revealed that TTC9A (1–95), TTC9A (1–105) and TTC9A (1–110), which include the first TPR domain, showed obvious interaction with Tm5NM-1. In addition, the linker peptide (aa 91–127) between the first and the second TPR may facilitate the binding between these two proteins, as TTC9A (1–128) pulled down more Tm5NM-1 than TTC9A (1–95), TTC9A (1–105) and TTC9A (1–110) did (Fig. 4C).

The observation that full-length TTC9A showed weaker interaction to Tm5NM-1 than TTC9A (1–115) and TTC9A (1–128) (Fig. 4B and 4C) suggested that the C-terminal part of TTC9A protein could have some inhibitory effect on the interaction between these two proteins (Fig. 4B and 4C). This notion is further supported by the observation that TTC9A (1–161) and TTC9A (1–197) pulled-down less Tm5NM-1 than TTC9A (1–128) did (Fig. 4C).

To further verify the importance of TTC9A (1–50), the linker region and the second and the third TPR domains in the interaction with Tm5NM-1, an experiment was performed to take account of the amount of TTC9A and the truncated proteins pulled-down (Fig. 4D). It shows that TTC9A (1–50) did not interact with Tm5NM-1 but TTC9A (51–115) did, even though similar amount of TTC9A (1–50) and TTC9A (51–115) were pulled-down. This suggests that the first TPR played some role in the interaction with Tm5NM-1. Secondly, although more TTC9A (51–222) (the saturated whitish band pointed by arrow) were pulled-down than TTC9A (51–115), it did not interact with Tm5NM-1 but TTC9A (51–115) did. We speculate that either aa.1–50 is required for the full length TTC9A to interact with Tm5NM-1 or that the second and the third TPR domain adversely affected the interaction. Although there appeared to be more TTC9A (1–115) pulled-down than TTC9A, the relative amount of Tm5NM-1 pulled-down by TTC9A (1–115) appears to be more than that by TTC9A, and this lends support to the speculation that the second and the third TPR domains are inhibitory to the interaction with Tm5NM-1. This takes account of the fact that the signal for the TTC9A band is saturated (Fig. 4D, lane two in the lower panel), so the amount of TTC9A pulled-down is more than it appears to be.

It is very important to verify the interaction between endogenous TTC9A and Tm5NM-1. We have tried several times for the endogenous pull-down using either TTC9A or Tm5NM-1 polyclonal antibodies. Unfortunately, we obtained very weak pull-down of high background. We suspect that the binding of polyclonal antibodies to multiple epitopes of the endogenous TTC9A or Tm5NM-1 interfered with their interaction with the target protein. Regrettably, monoclonal antibodies to TTC9A or to Tm5NM-1 are not available at this point in time.

Non-muscle tropomyosins generally help to stabilize actin filament. Over- expression of tropomyosin-1 in breast cancer cells MDA-MB-231 was found to promote the assembly of stress fibers [40]. The interaction of TTC9A with Tm5NM-1, together with the observation that in breast cancer cell line ABC28, TTC9A was up-regulated by progesterone, accompanied with a drastic increase in focal adhesion and in F-actin formation [41], led us to hypothesize that TTC9A may be involved in cell cytoskeleton organization and cell adhesion. However, knock-down of TTC9A expression by 70 – 80% did not abolish progesterone-induced increase of F-actin (data not shown). This suggested that either TTC9A was not essential in the formation of focal adhesion and stress fibers, or that other TTC9 family proteins were able to compensate for the lost function of TTC9A.

Tm5NM-1 and other tropomyosin family members are well-known for their association with the cytoskeleton system. An elevated level of Tm5NM-1 has been found in high-metastatic mouse melanoma cells and transformed rat fibroblastic cells, which suggested a function of Tm5NM-1 in inhibiting the polymerization and/or the formation of the bundles of actin microfilaments [35,36]. Studies have also revealed that the multiple isoforms of non-muscle tropomyosin might play a role in modulating the organization of microfilaments in cells by regulating the interaction between actin and other actin-binding proteins, such as filamin, spectrin, caldesmon, gelsolin and DNase I [42-44]. Thus, it is possible that TTC9A participates in the complex cytoskeleton regulation through its interaction with Tm5NM-1, or with other tropomyosins.

## Conclusion

In summary, this study revealed that TTC9A was over-expressed in breast cancer tissues compared with the adjacent normal tissues, suggesting that TTC9A might be an important gene involved in the breast cancer development process. We have identified Tm5NM-1, a tropomyosin family protein, as one of the TTC9A-interacting proteins. The results also suggest that the first 50 aa of TTC9A was required for the interaction with Tm5NM-1, although the segment alone did not bind to Tm5NM-1. Furthermore, the first TPR domain and the linker segment between the first two TPR domains may play an important role for the binding of TTC9A to Tm5NM-1, while the last two TPR motifs may be inhibitory on the interaction. The exact function of TTC9A remains unknown at current stage. The interaction with Tm5NM-1 suggests that TTC9A might act as a chaperone protein in the organization of cell cytoskeleton. Currently, TTC9A gene knockout study in mice is underway to define the physiological role of the gene *in vivo*.

## Competing interests

The authors declare that they have no competing interests.

## Authors' contributions

SLC performed most of the experiments, analyzed and interpreted data and participated in the manuscript writing. GHH provided the tissue samples and conducted clinical analysis of the data. VC–LL designed the experiments, acquired financial support and participated in manuscript writing. All authors have contributed to this work, read and approved the final manuscript.

## Pre-publication history

The pre-publication history for this paper can be accessed here:


